# The novel narrative technique uncovers emotional scripts in individuals with psychopathy and high trait anxiety

**DOI:** 10.1371/journal.pone.0283391

**Published:** 2023-03-23

**Authors:** Barbara Gawda

**Affiliations:** Department of Psychology of Emotion & Personality, Maria Curie-Sklodowska University, Lublin, Poland; University of Rome La Sapienza: Universita degli Studi di Roma La Sapienza, ITALY

## Abstract

Mental representations are of great importance for understanding human behaviour. The aim of this article is to present an innovative way to assess emotional scripts, which are a form of mental representations of emotional events, based on an analysis of narratives and their contents. Theoretical background on emotional schemas and scripts is provided along with information about types of related measures. Then, a rationale is presented for introducing an assessment of scripts related to specific emotions such as love, hate, and anxiety in a psychopathological context. This is followed by a perspective explaining the procedure of the relevant technique based on narrative data analysis. The technique has been successfully applied in two studies [I study (*n*– 200), II study (*n*– 280)]. A total of 1440 narratives about specific emotions have been analyzed to identify the indicators of scripts. The psychometric properties of the proposed technique have been established such as reliability, inter-rater agreement, and accuracy. The results show the value of the assessment of emotional script in individuals, particularly with high psychopathy and high trait anxiety. The contents of love and hate scripts are an illustration of cognitive distortions and deficits in the emotional information processing in individuals with psychopathy. The method enables the collection of informative data on romantic love, hate, and anxiety scripts which provides insight into how people may perceive and experience emotions and how they behave emotionally. Future research should focus on verification of the technique in other types of psychopathology and on the improvement of computer software dedicated to the narrative technique described in this paper.

## Introduction

The purpose of the paper is to introduce and describe an innovative method focused on analysis of scripts of specific emotions such as romantic love, hate, and anxiety. These emotions are of key-importance in explanation of behaviour in terms of clinical/psychopathological functioning. The technique is based on analysis of the content of narratives and let’s to predict emotional deficits based on the description of the elements of emotional scripts. The novel technique and the concept of emotional script have been elaborated on the bases of cognitive and narrative theories of emotions. According to this theoretical background, emotional script is defined as a form of generalized mental representations of specific emotional events [1, 2]. The narration about an emotional event contains narrative schema which can reflect emotional script [3]. The narrative technique was developed and described in this paper is a type of photo elicitation technique. This technique uses similar initial stage of the testing as the Picture Story Exercise/TAT or other PSE variants, i.e. procedure of eliciting emotions/motives. Persons are instructed to imagine and write a story after seeing the picture [2, 4, 5]. However, the narrative technique of assessment of specific emotional scripts presented here is different than the PSE, it is based on another theoretical approaches i.e. the narrative and psycholinguistic approaches to emotions. These assume that the narrations contain narrative schemas which can reflect emotional schemas [1, 3]. Then, the procedure of examining of narrative material is different from this in the PSE, it is focused on specific emotional scripts.

### The rationale for examining the specific emotional scripts

Although the existing methods and techniques of script examination may be useful for personal, life or interpersonal affective script assessment, they are not focused on assessing scripts related to specific emotions. In the context of psychopathology, it is important to examine specific emotions, such as hate or anxiety, because people with some disorders do not display general emotional deficits but specific emotion impairments, e.g. they have problems with anger. The example of a technique dedicated to specific emotion schemas is the Hatfield-Rapson Love Schemas Scale focused on the examination of love. However, this is a self-report measure and its application in psychopathology is limited because of the potential inaccuracy associated with an impaired capacity for insight in people with mental disorders [6]. Assessment of specific emotional scripts may enable better understanding of human behaviour incoherencies/dysfunctions. Importantly, psychotherapy clients commonly hold multiple, rather than just one, interpersonal scripts [7]. Furthermore, specifically targeted analyses revealed that people with some disorders differ in experiencing particular emotions, e.g. individuals with antisocial personality disorders display significant impairments in love, and closeness, but not in anger [8]. They present specific impairments in anxiety (particularly in cognitive anxiety rather than physiological), but not in hostility [9]. Given this, it would be worthwhile to test a specific emotional script and not the general interpersonal or life scripts like those tested by FRAMES or QUAINT [10]. Moreover, the FRAMES and QUAINT are valuable tools enabling examination of personal scripts, such as those related to trait-like phenomena, but they are not dedicated to specific emotions; they are based on autobiographical narratives about emotional situations in general [10]. Hence, narrative analysis focused on a general tendency could identify characteristics of affectivity but it would be inaccurate in describing schemas of specific emotions. That is why the novel technique of examining the affective schema related to specific emotions in individuals with personality (or other) disorders may be of great importance.

It is noteworthy that the novel technique proposed in this paper is not appropriate for assessing an experience of emotions (polysemantic factor). Emotional scripts are not the same as emotion experiencing, however, they are associated. Research indicates that people differ in the way they experience love, hatred, and anxiety, which is linked to the existence of different schemas/scripts of these emotions [11, 12]. This is especially pronounced in psychopathology.

The approach and technique proposed here are dedicated specifically to the description of the scripts of three emotions, i.e. romantic love, hate, and anxiety. The three examined here emotions seem to be particularly problematic in psychopathology [13]. Love is an emotion playing fundamental role in human relationship and in individuals with psychopathy or/and antisocial personality is dysfunctional [14]. This group of persons present high tendencies to experience emotions from the hatred spectrum [8, 14]. Hate particularly influences relationships among antisocial/psychopathic offenders [8, 14, 15]. As regards to anxiety, there are people do not seem to experience different types of anxiety, such as those with antisocial personality disorder and/or psychopathy, or individuals who have more developed anxiety scripts, such as patients with bipolar disorder [13, 14]. Anxiety is associated with almost all types of psychopathology. Psychopaths experience anxiety in a non-adequate way to stimuli [8]. Thus, to describe and explain their difficulties it is worthy to examine them. The reported in this paper results are part of a larger project focused on many disorder types. Psychopathy was chosen as one of the examined disorders as in this disorder emotional problems in romantic love, hate, and anxiety are particularly pronounced. Individuals with psychopathy display difficulties in closeness (romantic love), they experience excess hatred, and impaired anxiety (particularly cognitive anxiety) [8, 14, 16].

### Emotional schemas and scripts as mental representations

The term ‘emotional script’ will be used this paper. This author’s concept of ‘emotional scripts’ was elaborated as it illustrates well the specific emotion representation content. The author’s concept of ‘emotional script’ is based on the approaches proposed by Crits-Christoph, Demorest, Muenz, and Baranackie [7], Demorest and Alexander [17], Schank and Abelson [18], Schweder [11], and Sternberg’s *love is a story* concept [12]. **The author’s approach refers to structural aspect of script and assumes that emotional scripts are thought as to be mental representations of specific emotions. The components of an emotional script are the following: number of positive (or negative) descriptions of an actor, number of negative positive (or negative) emotions of the actor, number of positive (or negative) descriptions of a partner, number of positive (or negative) emotions of the partner, actions between the actor and the partner, evaluation of the importance of a scene, type of ending of a scene. Such scripts** do not refer to a wide range of life situations but to particular emotional situations such as romantic love, hate, or anxiety events, they reflect people’s personal knowledge on emotional events, they encode/contain information on how people understand and experience the specific emotions as Schweder stated [11]. Furthermore, emotional scripts store basic and abstract knowledge related to emotional events/emotional states [10, 17]. The emotional scripts store an interpretation of specific situations, not just their reproduction. These scripts can encompass individual/personal elements (events specific to a given person i.e. betrayal, resentment) which were highlighted by Crits-Christoph, Demorest, Muenz, and Baranackie [7]. These individual events can be part of the emotional scenario. Emotional scripts are similar to mental scenes/scenario and they can contain the elements of a typical scene such as scene sequence, characters (actors), propos, scenario conditions, and results as proposed by Schank and Abelson [18, 19]. This approach also assumes that narrative analyses allow to describe the content of emotional scripts because narratives contain narrative schemas which can reflect cognitive or emotional schemas [1].

Noteworthy, in the literature, researchers use different terms that are similar to scripts to name mental representations of emotions. The mostly known terms are *schemas* and *working models*. The history of these terms and other similar to them were thoroughly analysed by Demorest [10]. All of them are forms of cognitive generalizations that contain crucial information about emotional functioning. *Working models* is a term which refers to all types of mental representations including emotional ones, it has a largest meaning. A term *schema* is earlier, it was established by Piaget and Bartlett in connection to cognitive development and later implemented in personality psychology [10, 20, 21]. The terms *schema* and *script* are defined similarly, however scripts are thought to be schemas of event; generalized event representation [10]. The researchers argue that schemas and scripts are formed as a result of information processing involving multiple repetitions of events, social influence, personal wishes, aims and past experience [10, 20]. Scripts and schemas can be analysed with regard to structural aspects (the content of a script/schema) and functional aspects—they are a cognitive framework for organizing, interpreting, and recalling information [17]. The functional aspect was pointed out by Schweder [11] who defined scripts as interpretive schemes which provide a routine or plan for making sense of emotional experience in ways which are meaningful to the individual. In terms of functional aspect the emotion scripts are “like working models’ according to Saarni [22].

In general, the approaches to schemas and scripts can be divided into two main groups of defining emotional schemas/scripts.

1. First group, schemas/scripts are believed to be general dispositions, general tendency to interpret affective events which means that they refer to a large set of situations (they are trans-situational such as life scripts or general personal scripts). According to this group of approaches, they are similar to traits or trait-like phenomena that organize emotional experience and emotional processing [23]. They are a kind of mental representations and affective-cognitive structures [10]. The example of this type of scripts can be *secure base script* which is general, i.e. it refers to wide range of life situations [21]. Another example of this type of defining can be Tomkins’s [24] concept of a script (also named as a personal script) which is understood as a general tendency to interpret and organize affects and to behave in a particular way. As an example, scripts defined this way include such life scripts as *macho* script and *nuclear scripts* proposed by Tomkins. In this main group of defining emotion schemas are thought to be either adaptive or maladaptive. Adaptive schemas simplify the mental process of information analysis, provide rapid deployment of cognitive strategies, and improve information processing while maladaptive schemas may lead to problems in perceiving novel situations [13, 17]. A number of concepts for maladaptive schemas in psychopathology have been developed in theories by such authors as Beck, Leahy, and Young. As an example, schemas defined in this way (i.e. general traits) are illustrated by Leahy’s *simplistic view*, or Young’s *grandiosity* schema [25, 26].
2. The second group of approaches to emotional scripts was mentioned at the beginning of this part of the paper, i.e. emotional scripts are mental representations of the specific emotional events such as romantic love. They are not defined as general disposition, i.e. they do not refer to wide range of different emotional situations but to specific emotions.

Romantic love. With regard to romantic love, theoreticians argue that individuals own different love schemas or cognitive models of love that are inter-correlated [12]. A cognitive theory of romantic love by Sternberg and Weis [27] assumes that love is a stable and complex attitude, and people can have varied concepts related to that. The theory named *love as a story* shows that narrative about romantic love contain information about actors (and partners). Although Sternberg do not uses terminology as actor, valence or types of actions, his analyses and typology of love stories produced by people allow to indicate several characteristics expressed in love stories. These are: valence of emotions, actions that can be divided into several types, i.e. actions towards the other, actions away from the other, and scene endings. The variety of romantic love forms expressed in the narratives is determined by variety in love scripts [28].

Hate. The second crucial emotion from human relationship, particularly in psychopathological context, is hate. As for hate scripts, they seem to comprise extremely negative elements such as negative perception of the object of hate, negative causes, negative actions, and consequences [29, 30]. The three-component conception of hate by Sternberg [30] points out the different types of hate. It suggests that there are different hate scripts. Baumeister and Butz [31] showed two important mechanisms of hate that allow to understand the varied working models of hatred. For instance, hate schemas are thought to be frequently activated in antisocial individuals who were tested with the use of the narrative analysis [32].

Anxiety. Another important specific emotion is anxiety. It can be experienced in the absence of a direct threat and lasts longer than fear [33]. With regard to links between anxiety and psychopathology, there are a lot of conceptions which consider anxiety to be important in explaining the mechanisms of various disorders. The existing differences in experiencing anxiety suggest that people have different anxiety scripts, i.e. there are people who have less developed scripts of anxiety, such as those with antisocial personality disorder and/or psychopathy, or individuals who have more developed anxiety scripts, such as patients with bipolar disorder. Thus, the research on the anxiety schemas provides important insights into psychopathology of personality [34, 35].

### Measures of schemas/scripts

The existing methods for assessing emotional scripts or schemas can be either idiographic or nomothetic. Among nomothetic methods, a number of inventories to assess emotion scripts/schemas have been developed. They are designed to measure maladaptive schemas because of their significant role in psychopathology such as depression, anxiety, personality disorders, aggression, substance abuse [25, 26, 34]. An example of self-report scale is the Young Schema Questionnaire (the YSQ-SF, the SQC, or the YPI) and the LESS by Leahy [25, 34, 36, 37]. (The example of a question in the Leahy Emotional Schemas Scale; question no 44: “*I worry that if I have certain feelings I might go crazy*”). These are valuable, quick, and simple measures. Strong convergent and discriminant validity, as well as other strong psychometric properties have been demonstrated for them [37]. However, being self-report techniques, they all have limitations typical for self-measures; these are particularly prominent in psychopathology in examination of individuals with impaired insight [36, 37].

The second group of the method i.e. idiographic methods of assessing scripts, are of a special value in clinical assessment [38]. Their advantages include the fact that they lead to full understanding of a person’s uniqueness and complexity, but on the other hand, they are less suitable for empirical research, since no standard categories or quantitative ratings are available [38, 39]. Saarni proposed measurement of emotion scripts in children by observation of a child’s behaviour when social expectations are violated; this includes event sampling, and collecting of narratives. The observation and event sampling techniques allow to identify emotion scripts, yet they do not enable description of their contents or potential regulative role [22]. Unlike event sampling and observation, narrative was suggested as a valuable tool enabling description of script content. In this case analyses are based on the assumption that narratives embody scripts, and that narratives and scripts are structurally similarly [39]. This standpoint resulted in the development of narrative techniques for script/schema assessment [38, 39]. It seems that the mixed approach combining idiographic and nomothetic views is the most informative. In this context, the tool called FRAMES (Fundamental Repetitive and Maladaptive Emotion Structures) was developed for measurement of interpersonal schemas [39]. Its advantages include the fact that the FRAMES reveal the maladaptive affective scripts manifested in stories. The tool allows to connect idiographic scripts with nomothetic content and objective testing of changes in maladaptive patterns. The FRAMES have certain disadvantages, as this is a time-consuming and labour-intensive method of assessment [39]. Similarly, the QUAINT (Quantitative Assessment of Interpersonal Themes) was developed to facilitate empirical research on scripts [40]. This technique focuses exclusively on interpersonal scripts. It is based on autobiographical narrative analyses. The QUAINT system has proved to be a fruitful method in psychopathology and psychotherapy and shows several advantages, such as the ease of use, and the capacity to quantify scripts. However, it has some limitations, namely the complexity of the coding system makes it difficult to achieve good inter-rater reliability. Furthermore, the system for script coding was derived from samples of college students [40, 41].

### Research aims

The purpose of the paper is to introduce a method for emotional scripts assessment. Two studies were performed. The study I was undertaken to identify and describe the content of romantic love, hate, and anxiety scripts through the narrative analyses. Then, it has been checked whether the identified scripts’ indicators are inter-correlated (separately for each emotion), and whether inter-rater reliability and internal consistency are sufficient. The purpose was also to show discriminant validity of the technique through checking whether emotions scripts’ patterns are different in persons diagnosed with psychopathic personality disorder from those without this disorder. Next, study II was conducted to confirm the configurations of script’ patterns that was identified in the Study I. These two studies were undertaken to establish the psychometric properties such as reliability (inter-rater reliability, test-retest) and validity of the proposed method of romantic love script, hate, and anxiety scripts assessment. With regard to evidence predictive validity of the technique the multiple regression analyses have been conducted (separately for each emotion and in total emotions together), that aimed to show whether script’s components let to predict emotional impairments and psychopathology, in particular, what scripts’ patterns allow predicting psychopathic personality traits and state/trait anxiety. These forms of psychopathology described in this paper are the examples from the larger project focused on many disorders.

## Materials and methods

### Ethics statement

The participants gave the written consent to participation in the present study according to the guidelines approved by a local Ethics Committee of University of Maria Curie-Sklodowska (no of the protocol 2019/06). All necessary permissions for the study in prisons have been obtained.

### Pilot study

Initially, a pilot study was conducted. A sample of 40 persons (50% of men, 50% of women), with different education levels, voluntary participated in the study. They were asked to look at eight photographs (selected prior by four competent judges/psychologists) shown separately. Before examination of the pilot sample of 40 persons, three competent judges were shown 30 photos of emotional situations of love, hate, and fear (10 photos for each emotion). They were asked to select photos that they thought best show emotions of love, hate, and then, fear. The criterion was to determine which photos reflect /express love, hate, and fear best. The judges could choose as many photos reflecting each emotion as they wanted. They chose a total of 8 photos: 3 showing love, 2 for hate, and 3 for fear. Then, these eight photos were presented to a pilot sample, 40 people were asked to say what emotions are expressed by the photographs presented to them. The participants’ descriptions of each picture were open-ended, they should name any emotions they wanted. Then, participants should rate i.e. say to what extent the picture reflects romantic love, hate, anxiety or other emotion (if they named other emotions). Participants performed these tasks separately for each emotion. They rated the photographs on a 3-point scale (1 –“slightly agree”; 2 “agree at some point”; 3 “strongly agree”). Three photographs receiving the most significant value (W- Kendall test = .93 for love photograph, W = .86 for hate photograph, W = .88 for anxiety photograph) were chosen for the next stage of the investigation. Pictures of emotional situations used in the narrative technique were presented in the book published in 2007 [42].

## Study I

### Participants

The study involved a sample of 200 subjects (men) aged between 18 and 50 years heterosexual adults. This sample included three groups: prisoners with high psychopathy (*n* = 50), prisoners without psychopathy disorder (*n* = 50), non-prisoners (*n* = 100). Prisoners have been chosen as the participants for the study because of potential high occurrence of antisocial personality disorder and other personality disorders in this population [43]. This population is also typical in terms of high level of emotional psychopathology. The diagnoses of the prisoners were established by clinicians and found in their dossier (psychopathy was assessed with the use of the PCL-R and clinical analysis of behaviour). The participants were able to opt out of the study. The prisoners who did not participate in the study received the same treatment as study participants. Three groups were matched in terms of intelligence (the WAIS-R; IQ = 90–110); all demographic data are presented in Table 1 and other characteristics such as age, or lack of psychiatric, neurological, or somatic disorders (demographic and other data based on the screening questionnaire). They completed narrative tasks, WAIS-R, and other questionnaires. In total, they produced 600 narratives (200 about romantic love, 200 about hate, and 200 about anxiety) that have been analyzed. Descriptive statistics for narrative variables are presented in Table 2.

**Table 1:** Comparisons between the groups (demographic, intellectual characteristics, length of narratives): Prisoners with high psychopathy scores, prisoners with low psychopathy scores, non-prisoners with low psychopathy scores (*n* = 200).

Variables	Prisoners with high PS	Prisoners without PS	Non-prisoners	*F*_*(2*, *197)*_
	*n* = 50	*n* = 50	n = 100	
	*M(SD)* ^*a*^	*M(SD)*	*(M*, *SD)*	
**Age**	34.2 (10.00)	34.4 (9.00)	33.9 (8.80)	1.25
**Education**	10.17 (1.80)	10.40 (1.50)	10.44(1.50)	.66
**Vocabulary**	44 (8.00)	45 (7.00)	45 (7.00)	1.04
**Psychopathy**	33.03 (4.07)	23.07 (2.74)	17.12 (3.14)	202.17***
**Length: love**	34.44 (6.25)	36.02 (3.32)	31.41(4.33)	11.28***
**Length: hate**	31.19 (4.40)	18.75 (3.31)	18.74 (9.03)	14.16***
**Length: anxiety**	28.96 (6.78)	28.00 (3.74)	23.97(3.14)	1.76

^*a*^*M–*mean, *SD–*standard deviation

*** *p* < .001.

**Table 2:** Descriptive statistics: For love, hate, anxiety scripts (*n* = 200).

Narrative indicators	Min.	Max.	M^a^	SD
**Love Actor negative**	2.00	4.00	2.16	.49
**Love Actor positive**	2.00	8.00	4.56	1.64
**Love Actor negative emotions**	2.00	4.00	2.35	.60
**Love Actor positive emotions**	2.00	8.00	4.56	1.64
**Love Partner negative**	3.00	4.00	3.08	.28
**Love Partner positive**	3.00	6.00	3.74	.95
**Love Partner emotions negative**	2.00	3.00	2.98	.11
**Love Partner emotions positive**	3.00	6.00	3.53	.86
**Love Actions towards**	3.00	5.00	3.65	.71
**Love Actions from**	2.00	4.00	3.03	.24
**Love Actions against**	2.00	3.00	2.99	.07
**Love Important**	3.00	5.00	3.52	.71
**Love Unimportant**	2.00	3.00	2.98	.11
**Love Positive ending**	3.00	4.00	3.68	.46
**Love Negative ending**	3.00	4.00	3.23	.42
**Hate actor negative**	1.00	5.00	1.68	1.16
**Hate actor positive**	1.00	3.00	1.41	.74
**Hate actor emotions neg**	1.00	5.00	2.11	1.07
**Hate actor emotions pos**	1.00	3.00	1.22	.59
**Hate partner negative**	1.00	2.00	1.36	.48
**Hate parner positive**	1.00	2.00	1.00	.07
**Hate partner emotions neg**	1.00	2.00	1.48	.50
**Hate partner emotions pos**	1.00	2.00	1.00	.07
**Hate towards**	1.00	5.00	1.96	1.22
**Hate from away**	1.00	2.00	1.73	.44
**Hate against**	1.00	2.00	1.15	.35
**Hate Important**	1.00	2.00	1.14	.35
**Hate Unimportant**	1.00	3.00	1.13	.46
**Hate positive ending**	1.00	2.00	1.43	.49
**Hate negative ending**	1.00	2.00	1.38	.48
**Anxiety actor negative**	1.00	7.00	2.94	1.86
**Anxiety actor positive**	1.00	2.00	1.08	.28
**Anxiety actor emotions neg**	1.00	7.00	2.99	1.81
**Anxiety actor emotions pos**	1.00	2.00	1.08	.28
**Anxiety partner negative**	1.00	3.00	1.24	.55
**Anxiety partner positive**	1.00	2.00	1.05	.23
**Anxiety partner emotions neg**	1.00	3.00	1.45	.74
**Anxiety partner emotions pos**	1.00	2.00	1.09	.07
**Anxiety towards**	1.00	2.00	1.41	.49
**Anxiety from away**	1.00	2.00	1.05	.23
**Anxiety against**	1.00	2.00	1.01	.11
**Anxiety important**	1.00	2.00	1.43	.49
**Anxiety Unimportant**	1.00	2.00	1.07	.07
**Anxiety positive ending**	1.00	2.00	1.35	.48
**Anxiety negative ending**	1.00	2.00	1.48	.50

^a^ *M*–mean, *SD*- standard deviation.

The non-prisoners/controls have been recruited through advertisement and selected from the adult population in Poland. All participants were of Polish origin, they were not paid for their participation in the examination. All prisoners were tested in four state prisons in the psychological offices while examination of the control group took place in schools and at the University.

### Measures

- The PCL–R by Hare was used to assess psychopathy level. The PCL––R comprises 20 items scored on a three-point scale (0, 1, 2) according to the extent to which it relates to a given person. It is a semi-structured interview. The total score ranges from 0 to 40 and the score of 30 is used as the cut-off for diagnosing psychopathy [44].
- Vocabulary subtest from the Wechsler Adult Intelligence Scale- R. WAIS-R is a general test of intelligence—the Polish adaptation was used [45]. The test can measure four index scores and the full scale IQ. Vocabulary scores were considered in the two studies to compare the participants’ verbal skills due to the fact that creation of a narrative depends on verbal intelligence. Cronbach’s alpha in the study I for Vocabulary = .92.
- The State-Trait Anxiety Inventory (STAI). In this analysis the Trait/State Anxiety scores were considered in the statistical analyses to examine whether it is possible to predict state/trait anxiety basing on the scripts patterns. The Polish adaptation of the STAI consists of 20 statements describing emotional conditions. The psychometric properties as reliability and validity of the STAI are very good [46]. Cronbach’s alpha in the present study I is: for trait anxiety = .88, for state anxiety = .87.
- The narrative technique of assessment of love, hate, and anxiety scripts

A growing body of narrative and psycholinguistic research suggests that language analysis can provide insight into psychological processes, such as cognitive, emotional states and personality dispositions as well as psychopathological patterns [47–49]. The narrative technique (photo elicitation technique) was developed to assess romantic love, hate, and anxiety scripts.

#### Procedure of analysis of the narratives

**Fig 1:**
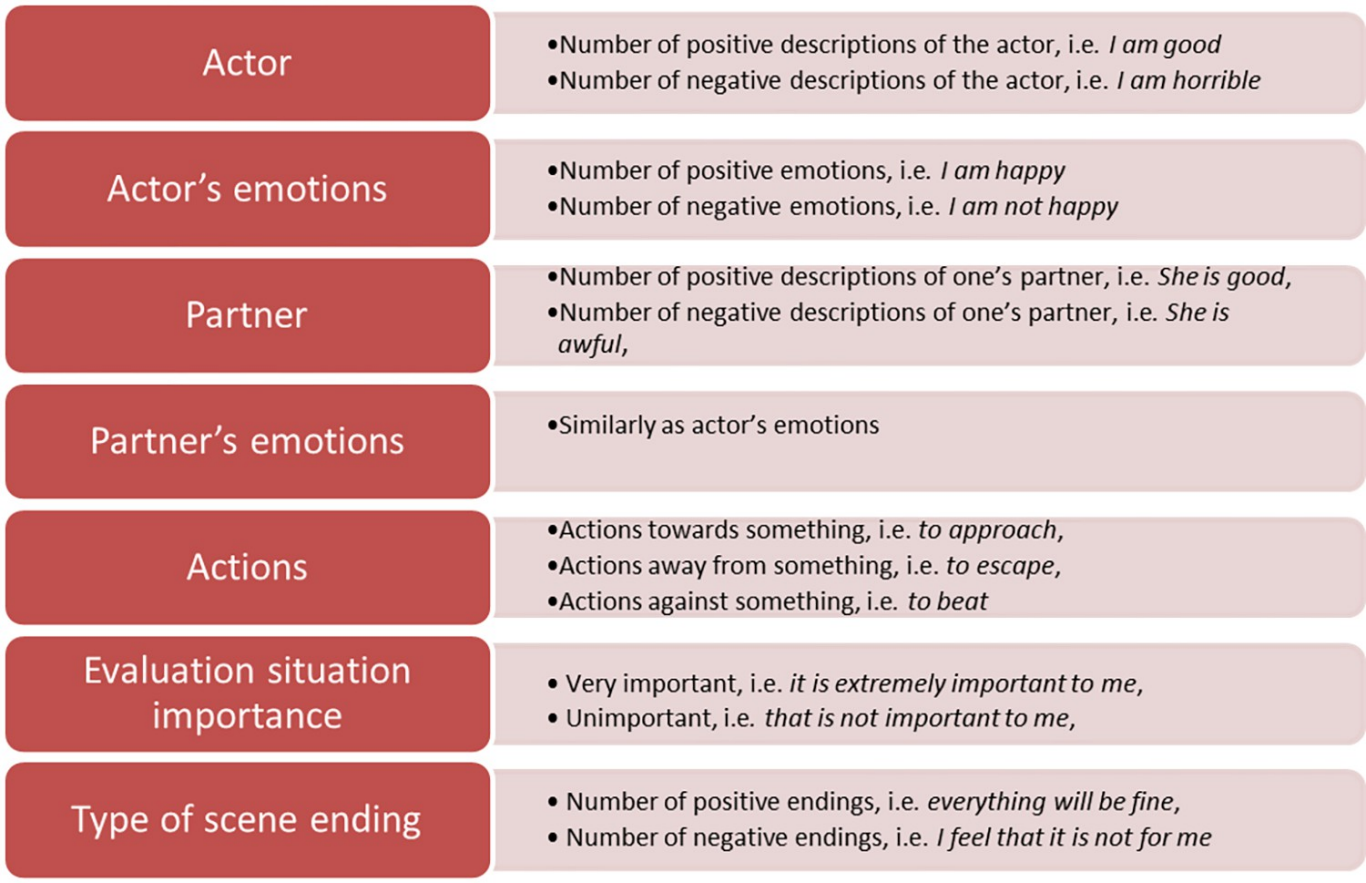
The components (and their indicators) of the scripts’ content.

Three photographs depicting love, hate, and anxiety, used as the main stimuli in the investigation, are presented in the book [42]. Each participant is asked to write one story about a situation involving love, hate, and anxiety (looking at the photographs). Each participant received the following instruction: “Take a look at the picture. Imagine that you are a person in the picture and write a story about it.” Then, stories were analyzed for the identified elements of emotional scripts (the indicators of emotional scripts are presented in Fig 1). The list of narrative indicators is elaborated on the bases of theoretical background and narrative theory of emotions, i.e. concept of emotional script which is defined as a mental representation of an emotional scene [11, 12, 18]. Emotional script as a scenario that can include as other types of script the elements proposed by Schank and Abelson [18] such as characters (actors), props, scenario conditions, results, and actions. The list of narrative indicators proposed by the author of this research and used in the present research contains: the number of positive (or negative) descriptions of the actor, the number of negative positive (or negative) emotions of the actor, the number of positive (or negative) descriptions of the partner, the number of positive (or negative) emotions of the partner, actions between the actor and the partner, evaluation of the importance of a scene, type of ending of a scene. These components describe the script content. All these indicators were numeric, i.e. were the number of words/statements/expressions. The narrative analyses performed by independent coders result in numeric scores (Table 2). This coding system was established based on a sample of adults representing the general population (participants from the study I and II), rather than college students.

*The analysis of narratives*: *procedure of identifying of the scripts’ components in narrative about love*

*Love script*. Here is a sample narrative (length: 101 words) about romantic love, written by a man with psychopathic personality disorder.

“*This is a picture when I felt really happy*. *I was ‘head over heels’ in love*. *I wish that time could return to me. Nothing else mattered but our love. When I am looking at the picture I feel my throat tighten. She has such joy in her eyes. She would constantly tell me she felt happy and safe with me. I was proud. I loved the whole world and I adored her joyful smile. This is the only picture I still have. I would do everything to snuggle close to her one more time. I was never so happy again.”*

Analysis of love narrative created by an individual diagnosed with psychopathy shows that they tend to use more positive than negative phrases related to themselves as protagonists (actors). The way of assessing the meaning of the event (evaluation) is noteworthy. Numerous phrases suggesting that this fact is extremely important are predominantly used by individuals with personality disorders. This is verbalised in the following way: “I felt really happy. . . I wish that time could return to me … I was proud …”, “I would do everything to snuggle close to her one more time. . .”. Story about love contain such definitive terms as *never*, *always*, *nothing*; they are meant to emphasise the importance of the situation. The story presents less elaborate characteristics of the partner/her emotions; the actor focuses on himself. The story also contains negative memories indicative of uncertainty, doubts related to the narrator’s role: “I was never so happy again”.

Elements of romantic love script in the sample narrative (all script indicators [see Fig 1] /variables were numeric, i.e. a number of words/statements/phrases were counted by the coders and provided as below):

- actor’s descriptions positive: 2, *I loved the whole world*, *I adored her*
- actor’s description negative: 0
- actor’s emotions positive: 5, the actor’s traits: *I felt really happy*, *I was proud*, *I was in love*, *I feel my throat tighten*, *I was ‘head over heels’ in love*
- actor’s emotions negative: 1, “*I was never so happy again*”.
- partner’s descriptions positive: 3, *She has such joy*, *she felt happy*, *safe*
- partner’s descriptions negative: 0
- partner’s emotions positive: 3, joy, felt happy, safe with me.
- partner’s emotions negative: 0
- activity “towards”: 2; remembering and recalling facts; *This is the only picture I still have*. *I would do everything to snuggle close to her one more time*
- activity from away: 0
- activity against: 0
- meaning/evaluation: 3—very high, *Nothing else mattered but our love*, *I was never so happy again*, *I feel my throat tighten*
- ending positive: 0
- ending negative: 1, *I was never so happy again*.

In order to objectively perform the narrative data analyses, a mathematical formula written in the Python programming language was used. It was designed to count words/phrases/statements. First, all words/phrases/statements associated with emotion scripts identified by the coders, as shown in the example above, were entered into the database of the software. Then, a special mathematical formula was applied to enable counting all the words representing the specified indicators. Subsequently, the narratives produced by a subject were uploaded into the software. As a result, the software produced numerical scores for all the types of indicators of emotion scripts that were programmed. Finally, the results were checked by the coders again for correctness. The coefficients of equivalence between the software scores and independent judges’ scores were very high (k ranges between .98 and 1.00).

## Results

### Reliability

#### The inter-rater reliability

The inter-rater reliability ([three blind and independent raters/coders—the Intra-class Correlation Coefficient] correlations ranging from .83 to .97) between the scores for coding was high. Reliability of the derived components of romantic love was confirmed. Similar values were obtained for components of hate and anxiety scripts (see S1 Table).

#### The internal consistency

**Fig 2:**
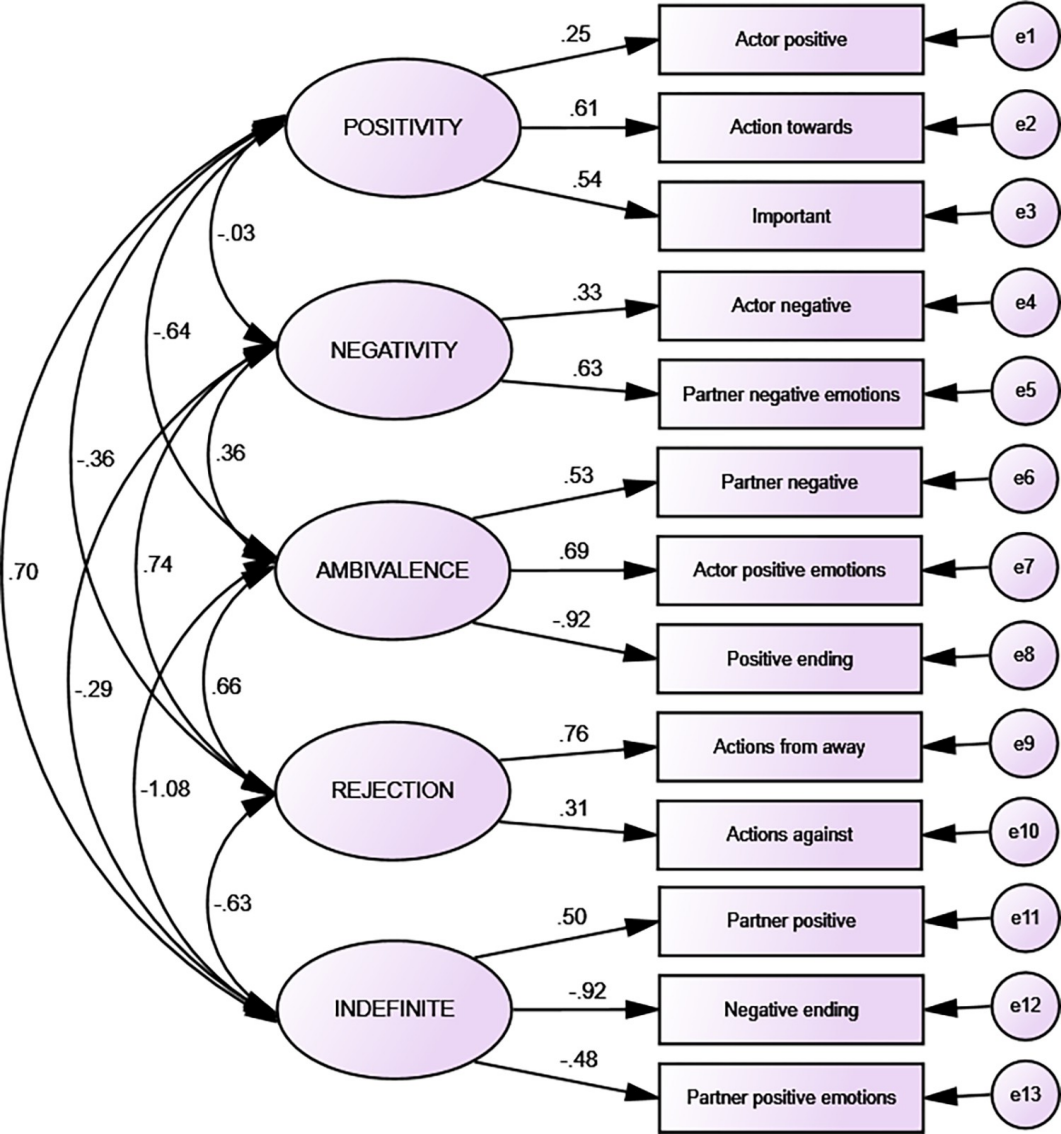
Love: Confirmatory factor analysis (*n* = 480).

**Fig 3:**
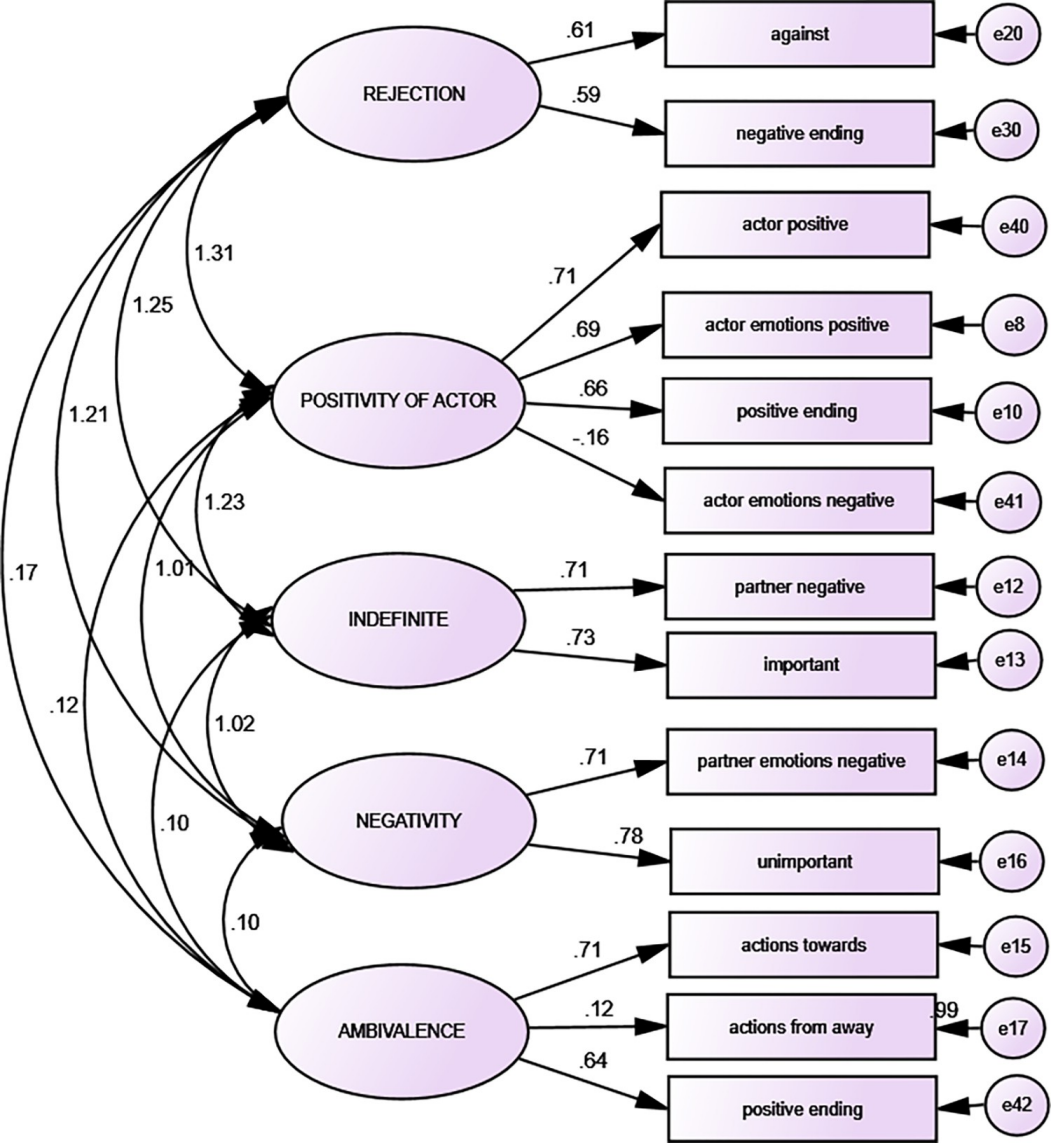
Hate: Confirmatory factor analysis (n = 480).

The internal consistency was tested. First, exploratory factor analyses were conducted on the sample of 200 people from Study I using Oblimin rotation separately for each emotion including (Figs 1–3 in S1 Fig include scree plots of EFA for each emotion is shown separately).

*Love script*. Exploratory factor analysis (EFA) revealed five main factors of love script (see Table 3) which explained 65.8% of variance (Kaiser-Meyer-Olkin Measure of Sampling Adequacy = .902, Bartlett’s test of sphericity = 1614.48; *p* < .001). The first factor called *positivity* was loaded by positive opinion about actor and about partner, positive emotions of actor, and importance of love. The second factor labelled *negativity* was loaded by negative opinion about actor and about partner, negative emotions of actor. The third factor *rejection* was loaded by actions against and unimportant evaluation. The fourth factor called *ambivalence* contains positive opinions on actor and on partner, negative emotions of actor, and positive ending of love stories. The fifth factor labelled *indefinite/unclear picture* contains positive opinions about partner, positive emotions of partner, actions towards, negative ending, and unimportant evaluation.

**Table 3:** Love: Exploratory factor analysis for narratives indicators (*n* = 200).

	Factors				
	Ambivalence	Indefinite	Positivity	Rejection	Negativity
**Actor negative**	-.439				.805
**Actor positive**			.966		
**Actor negative emotions**	.774				.432
**Actor positive emotions**			.916		
**Partner negative**	438		-.647		.496
**Partner positive**	.528	.841			
**Partner emotions negative**					
**Partner emotions positive**		.832			
**Actions towards**		.910			
**Actions from**					-.661
**Actions against**				.898	
**Important**			.558		-.539
**Unimportant**		.545		.927	
**Positive ending**	.895				
**Negative ending**	-.868	.404			

^a^Rotation Method: Oblimin with Kaiser Normalization.

*Hate script*. EFA revealed five main factors of hate script which explained 73.38% of variance (KMO = .822, Bartlett’s test of sphericity = 2033.51; *p* < .001). The first factor called *positivity of actor* was loaded by positive opinions about actor and positive emotions of actor unimportance of hate, and positive endings (EFA loadings are presented in Table 4). The second factor called *negativity* was loaded by low positive opinions about actor, negative opinions about partner, negative emotions of partner, and lack of actions towards. The third factor *rejection* was loaded by actions against and negative endings. The fourth factor called *ambivalence* contains positive opinions on actor, actions towards, actions from away, and positive endings. The fifth factor labelled *indefinite/unclear picture of hate* contains negative opinions about partner, positive emotions of partner, and importance of hate.

**Table 4:** Hate: Exploratory factor analysis for narrative indicators (*n* = 200).

	Factors				
	Ambivalence	Negativity	Indefinite	Positivity ofactor	Rejection
**Hate actor negative**	-.940				
**Hate actor positive**	.429	-.710		.453	
**Hate actor emotions neg**	-.921				
**Hate actor emotions pos**				.691	
**Hate partner negative**		.651	.508		
**Hate partner positive**					
**Hate partner emotions negative**		.888			
**Hate partner emotions positive**			.516		
**Hate towards**	.438	-.757			
**Hate from away**	.710				
**Hate against**					.892
**Hate important**			.849		
**Hate unimportant**				.850	
**Hate positive ending**	.655			.482	-.421
**Hate negative ending**	-.705				.521

^a^Rotation Method: Oblimin with Kaiser Normalization.

*Anxiety script*. EFA showed there are five main factors of anxiety script which explained 80.20% of variance (KMO = .782, Bartlett’s test of sphericity = 2012.22; *p* < .001). The first factor called *positivity* was loaded by positive emotions of partner and low number of actions against (EFA loadings are presented in Table 5). The second factor labelled *negativity* was loaded by negative opinions about actor and negative emotions of actor. The third factor named *rejection* was loaded by actions from away and lack of positive opinions on partner. The fourth factor labelled *ambivalence* contains positive opinions on actor, positive emotions of actor, negative emotions of partner, actions towards, and importance of anxiety. The fifth factor named *indefinite/unclear picture of anxiety* encompass negative opinions about partner, negative emotions of partner, importance, unimportance of anxiety, and positive endings.

**Table 5:** Anxiety: Exploratory factor analysis for narrative indicators (*n* = 200).

	Factors				
	Ambivalence	Negativity	Indefinite	Rejection	Positivity
**Anxiety actor negative**		.917			
**Anxiety actor positive**	.939				
**Anxiety actor emotions neg**		.910			
**Anxiety actor emotions pos**	.929				
**Anxiety partner negative**			.818		
**Anxiety partner positive**				-.939	
**Anxiety partner emotions neg**	.507		.765		
**Anxiety partner emotions pos**					.694
**Anxiety towards**	.539				
**Anxiety from away**				.979	
**Anxiety against**					-.630
**Anxiety important**			.589		
**Anxiety unimportant**			.589		
**Anxiety positive ending**			.710		
**Anxiety negative ending**		.779	-.425		

^a^Rotation Method: Oblimin with Kaiser Normalization.

### Validity

**Discriminant validity** of the technique has been tested. Significant differences were found in love, hate and anxiety scripts’ components between groups of prisoners with psychopathy, prisoners without psychopathy and healthy controls (see S2 Table). The differences between these type groups were partly suggested in the previous examination focusing on emotional language in persons with antisocial personality disorder [32].

#### Prognostic validity

Multiple regression analysis for love showed that love script’s patterns explain a significant amount of variance (23%) in Psychopathy (F_(5,194)_ = 9.34, *p* < .001). The significant predictors for psychopathic traits are ambivalence (β = -.34; *p* < .001), indefinite picture (β = .33; *p* < .001), rejection (β = -.12; *p* < .05), and positivity of actor (β = .17; *p* < .05) (see Table 6). Higher scores in indefinite picture and rejection, and lower in ambivalence and positivity of actor, are related to more notable psychopathic traits. Then, multiple regression analysis showed that love script’s patterns explain a significant amount of variance (7%) in state anxiety (F_(5,194)_ = 2.37, *p* < .05). The significant predictor for state anxiety is rejection in love script (β = .23) (see Table 7). Higher scores in rejection in love script are associated with higher state anxiety.

**Table 6:** Love script: Multiple regression analysis; script’ factors predict psychopathy.

Coefficients^a^					
Predictors	Unstandardized Coefficients		Standardized Coefficients	*t*	*p*
	B	*SE*	Beta		
**Love ambivalence**	-.782	.180	-.310	-4.338	.000
**Love indefinite picture**	.836	.182	.332	4.591	.000
**Love positivity**	-.322	.183	-.128	-1.862	.048
**Love rejection**	.317	.178	.126	1.975	.045
**Love negativity**	-.078	.180	-.031	-.432	.666

^a^Dependent variable: Psychopathy, *SE*–standard error.

**Table 7:** Love script: Multiple regression analysis; script’ factors predict state anxiety.

Coefficients^a^					
Predictors	Unstandardized Coefficients		Standardized Coefficients	*t*	*p*
	B	*SE*	Beta		
**Love ambivalence**	-.692	.750	-.073	-.923	.358
**Love indefinite picture**	.804	.758	.084	1.061	.290
**Love rejection**	2.146	.761	.225	2.819	.005
**Love positivity**	.733	.742	.077	.987	.325
**Love negativity**	.586	.751	.061	.780	.436

^a^Dependent variable: State anxiety, *SE*–standard error.

Multiple regression analysis for hate showed that hate script’s components explain a significant amount of variance (69%) in Psychopathy (F_(5,194)_ = 71.48, *p* < .001). The significant predictors for psychopathic traits are ambivalence (β = .22; *p* < .001), indefinite picture (β = .22; *p* < .001), rejection (β = -.39; *p* < .001), negativity (β = .57; *p* < .001), and positivity of actor (β = .08; *p* < .05) (see Table 8). Higher scores in ambivalence, indefinite picture and positivity of actor, and lower in negativity and rejection in hate script are associated with higher Psychopathy.

**Table 8:** Hate script: Multiple regression analysis; script’ factors predict psychopathy.

Coefficients^a^					
Predictors	Unstandardized Coefficients		Standardized Coefficients	*t*	*p*
	B	*SE*	Beta		
**Hate ambivalence**	.571	.117	.227	4.899	.000
**Hate negativity**	-1.447	.115	-.574	-12.598	.000
**Hate indefinite**	.544	.112	.216	4.867	.000
**Hate positivity of actor**	.215	.116	.085	1.856	.050
**Hate rejection**	-1.000	.115	-.397	-8.720	.000

^a^Dependent variable: Psychopathy, *SE*–standard error.

The example below of the story about hate well illustrates the above-mentioned patterns of hate script. The following story was written by a man with high psychopathy.

Sample narrative (length: 61 words):

“*Really*, *it’s nothing*. *Petty fight*. *I didn’t take out the garbage*. *She got mad*, *and I*. . . *I’m taking it easy*. *I’ll kiss her and we’ll make it up*. *All women are the same*, *they are always blaming someone else*. *But they are never the ones to blame*. *I’ll do everything to calm her down*. *I’m always easy-going and ready to agree*.”

Behaviours and personality traits of the character (actor, Fig 1) of the story about hatred are described in a rather interesting way. The man with antisocial personality disorder ascribes to himself such traits as amicability and kindness. The trait of amicability is verbalised for instance in the following ways in other narratives by individuals with this type of disorder: “. . .We will reach an agreement, everything will be all right. . .”, “I don’t like such situations, I always try to avoid them, I agree with the woman and I turn a quarrel into something joyful”, I’m taking it easy (actor’s emotions, Fig 1). . . “I avoid arguments, I own up to my mistakes even if haven’t made any. . .”. As a rule explanations take a definitive form: “such scene always ends with agreement” (type of ending, Fig 1). It is rather characteristic for individuals with diagnosed antisocial personality to more frequently use generalised phrases, such as “women always…” (partner, partner’s emotions, see Fig 1), “I always try to. . .”. As perceived by a criminal with antisocial personality, a scene of hatred, described by him, is unimportant (evaluation of situation, see Fig 1). Men with antisocial personality disorder far more often use hate phrases implying that this is something trivial and insignificant, e.g. “Petty fight, because I didn’t take out the garbage. . .”.

Elements of hate script in a sample narrative (a number of words or utterances or phrases were counted by the coders):

- actor / emotions, the actor’s traits/: from lack of emotions to feeling calm, *Really*, *it’s nothing*, *I’m taking it easy*.
- partner/emotions, the partner’s traits: negative perception, *She got mad*, *I’m always easy-going and ready to agree*.
- activity “towards”: *I’ll kiss her and we’ll make it up*, *I’ll do everything to calm her down*
- meaning: unimportant event, *Really*, *it’s nothing*. *Petty fight*
- generalization: *All women are the same*, *they are always blaming someone else*
- ending: positive, *I’m always easy-going and ready to agree*

Then, multiple regression analysis was performed to show whether hate script’s components predict trait anxiety. The analysis showed that hate script’s patterns explain a significant amount of variance (6%) in trait anxiety (F_(5,194)_ = 2.01, *p* < .10). The significant predictors for trait anxiety are rejection in hate script (β = -.22, p < .01) (see Table 9). Lower scores in rejection in hate script are associated with higher trait anxiety.

**Table 9:** Hate script: Multiple regression analysis; script’ factors predict trait anxiety.

Coefficients^a^					
	Unstandardized Coefficients		Standardized Coefficients	*t*	*p*
Model	B	*SE*	Beta		
**Hate ambivalence**	-.257	.700	-.030	-.367	.714
**Hate negativity**	-1.040	.690	-.121	-1.508	.134
**Hate indefinite**	.360	.671	.042	.536	.593
**Hate positivity of actor**	-.268	.696	-.031	-.384	.701
**Hate rejection**	-1.880	.689	-.219	-2.729	.007

Furthermore, multiple regression analysis for anxiety script showed that anxiety script’s components explain a significant amount of variance (45.9%) in Psychopathy (F_(5,194)_ = 25.59, *p* < .001). The significant predictors for psychopathic traits are ambivalence (β = .22; *p* < .001), indefinite picture (β = -.43; *p* < .001), rejection (β = .28; *p* < .001), negativity (β = .36; *p* < .001) (see Table 10). Higher scores in ambivalence, rejection, and negativity, and lower in indefinite picture of anxiety predict higher Psychopathy. Here an example of story about anxiety which is an illustration of the patterns of anxiety script in persons with psychopathic personality disorder.

**Table 10:** Anxiety script: Multiple regression analysis; script’ factors predict psychopathy.

Coefficients^a^					
Predictors	Unstandardized Coefficients		Standardized Coefficients	t	p
	B	*SE*	Beta		
**Anxiety ambivalence**	.557	.150	.221	3.714	.000
**Anxiety indefinite**	-1.104	.150	-.438	-7.353	.000
**Anxiety negativity**	.915	.150	.363	6.096	.000
**Anxiety rejection**	.719	.150	.285	4.790	.000
**Anxiety positivity**	.003	.150	.001	.021	.983

^a^Dependent variable: Psychopathy, *SE*–standard error.

The example below of the story about anxiety illustrates the above-mentioned patterns of anxiety script.

Sample narrative (length: 80 words) about anxiety written by a man with diagnosed antisocial personality disorder and Psychopathy:

“*Nobody notices me*, *I feel helpless and lonely*. *I feel ashamed to go out*, *because they will laugh at me*. *There were many such situations in my childhood*. *I am scared and terrified*. *I can see that my father is drunk and I’m afraid he will fight with my mom… But I feel calm*, *I know that in the future*, *when I am a father I will never allow for my child to look at life with such sadness*.”

The actor is the main character in the narrative; he perceives himself to be helpless and lonely, terrified and miserable. Generally, however, emotional ambivalence is characteristic for the actor’s feelings; on the one hand he is full of negative emotions, e.g. anxiety, uncertainty, sadness, shame, fear, helplessness and sense of loneliness; on the other hand he feels calm. It is possible to draw a conclusion that the story teller adequately identifies the cause for anxiety, and describes the characters (mainly himself in this case, but also other people involved in the scene) in a cohesive way. Other protagonists, in this case father, are described adequately (partner, partner’s emotions, see Fig 1). Yet, assessment of the situation (see Fig 1) reflected in the generalised belief seems to be characteristic. Emphatic use of the definitive terms *I will never allow* expresses dogmatism, and idealisation, and is accompanied with simultaneous contradiction (*I feel calm*) and strong personal involvement in the scene. The story lacks an ending, although the declaration regarding the future may be understood as a kind of conclusion.

Elements of anxiety script in a sample narrative (all variables were numeric, i.e. a number of words or utterances or phrases were counted by the coders):

- actor (emotions, the actor’s traits): *Nobody notices me*, *I feel helpless and lonely*, *I am ashamed*, *I am scared and terrified*, *I feel calm*
- partner/emotions, traits of the partner or other characters in the scene: *I can see that my father is drunk and I’m afraid he will fight with my mom*,
- activity “away from”: I feel ashamed to go out,
- meaning: highly important event, *There were many such situations in my childhood*, *I will never allow for my child to look at life with such sadness*
- ending: declaration, *I know that in the future*, *when I am a father I will never allow*

Then, multiple regression analysis was performed to show whether anxiety script’s components predict trait anxiety. The analysis showed that anxiety script’s patterns explain a significant amount of variance (8%) in trait anxiety (F_(5,194)_ = 2.82, *p* < .05). The significant predictors for trait anxiety are ambivalence (β = .19, *p* < .05) and positivity of anxiety script (β = .18, *p* < .05) (see Table 11). As higher positivity and ambivalence in anxiety script as higher trait anxiety is.

**Table 11:** Anxiety script: Multiple regression analysis; script’ factors predict trait anxiety.

Coefficients^a^					
Predictors	Unstandardized Coefficients		Standardized Coefficients	*t*	*p*
	B	SE	Beta		
**Anxiety ambivalence**	1.605	.661	.187	2.428	.016
**Anxiety negativity**	-.189	.661	-.022	-.286	.775
**Anxiety indefinite**	.792	.661	.092	1.198	.233
**Anxiety rejection**	.766	.661	.089	1.159	.248
**Anxiety positivity**	1.528	.661	.178	2.312	.022

^a^Dependent variable: Trait anxiety, SE–standard error.

Additionally, to show the global results, a multiple regression analysis including all emotions was performed. The results shows that the large model of regression included all emotional scripts’ factors (love, hate, and anxiety) as predictors explains about 84% of the variance of psychopathy scores. The details of this regression analysis have been presented in the S3 Table.

## Study II

### Participants

The study was designed to verify reliability of the linguistic method used in the study I. It is involved a sample of 280 subjects from general population (180 women, 100 men) aged between 19 and 50 years (M = 32.5, SD = 9.0), right-handed, heterosexual adults, who were selected to participate. They did not have any neuropsychiatric or somatic impairments, and were not addicted to drugs or alcohol (data based on the screening questionnaire). The education of participants was similar as in the study I (M _years of education_ = 10.54[in the Study I M = 10.17], SD = 1.36). They completed the same tasks as in the study I twice, i.e. they were reassessed four months later. In total, they produced 840 narratives (280 about love, 280 about hate, 280 about anxiety). Mean length of narratives about love, hate anxiety are presented in Table 1. Length of a story was measured by number of words in a story. Each narrative has been analyzed by three independent coders (psychologists) in terms of searching/identifying the components of emotion script.

Instruments used in the study II (the same as in Study I, described in the section Measures of Study I).

- The Vocabulary subtest from the WAIS-R. Cronbach’s alpha is .94.
- The State-Trait Anxiety Inventory (STAI). Cronbach’s alpha in the present study II is trait anxiety = .89, state anxiety = .87.
- The narrative technique of assessment of love, hate, and anxiety schemas (the same as used in the Study I)

## Results

### Reliability

#### The test-retest reliability

To establish test-retest reliability of the derived components of scripts, 280 subjects completed the task and then were reassessed four months later. Test-retest coefficients (Pearson correlations) are adequate, they range from .76 to .85 (see S4 Table).

#### The internal consistency

To confirm the configuration of scripts’ patterns revealed in the Study I confirmatory factor analyses were performed separately for each emotion (on the sample from study II, n = 280 and study I, total = 480). CFA for love was acceptable after removing some items loaded significantly more than one factor (data for love scripts: χ2/df = 836.19, CFI = .813, AGFI = .921; NFI = .871, RMSA = .069). CFA for love is presented in Fig 2.

CFA for hate characterizes acceptable parameters such as goodness of fit after not including some items which loaded not significantly factors or loaded more than one factor (data for hate scripts: (χ2 / df (15) = .634, CFI = .955, AGFI = .935, NFI = .904, RMSEA = .051)). CFA for hate is presented in Fig 3. CFA confirmed the fifth factor structure encompassing similar content as in EFA. However, there are some differences between the number of items (narrative indicators) loaded the factors comparing EFA and CFA.

**Fig 4:**
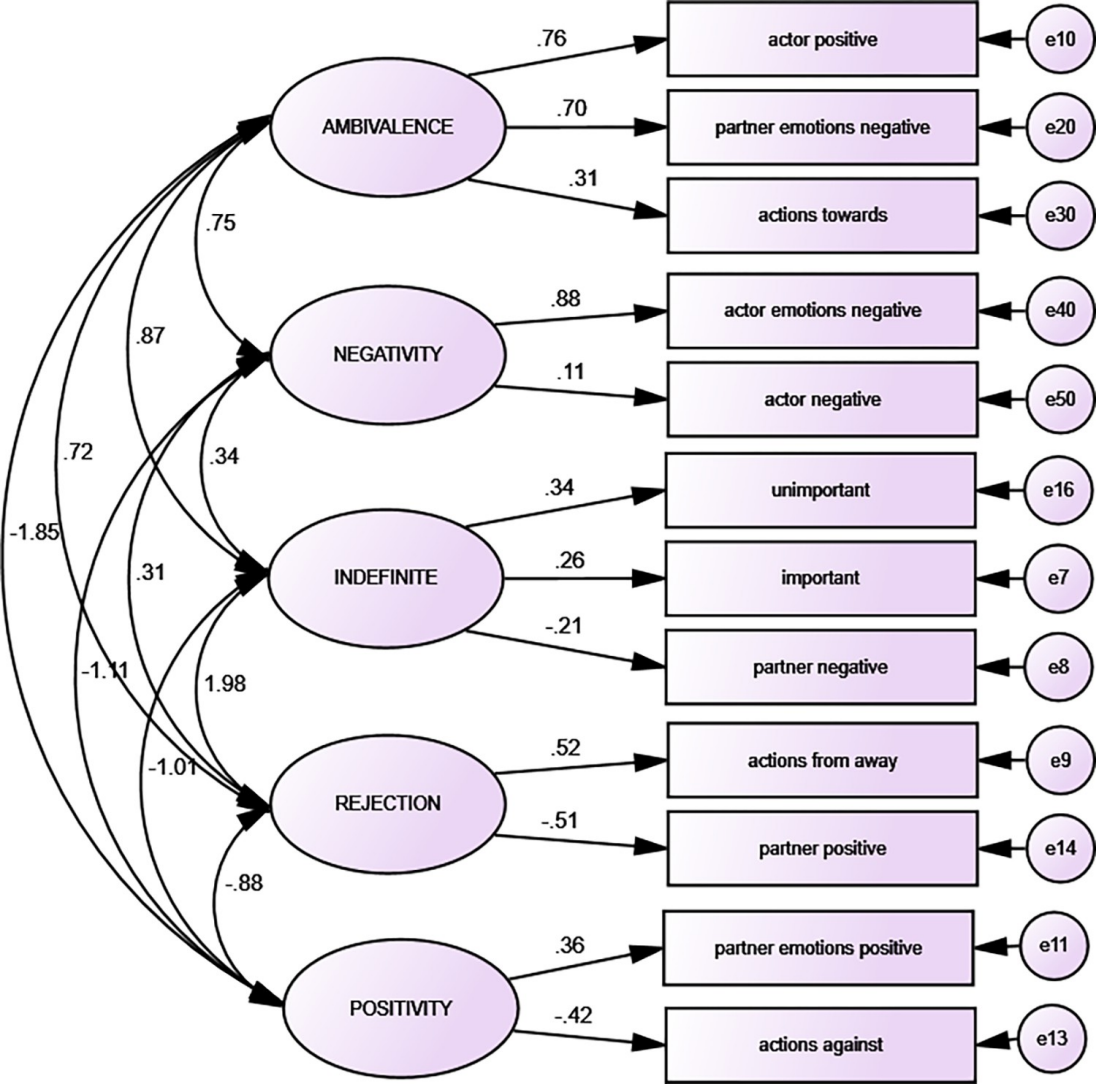
Anxiety: Confirmatory factor analysis (n = 480).

CFA results for anxiety are presented in Fig 4. CFA model is characterised by appropriate goodness of fit (χ2 / df (15) = .431, CFI = .955, AGFI = .921, NFI = .907, GFI = .970, RMSEA = .052). CFA confirmed fith’factor structure of anxiety script, however, there are some differences between numbers of items and loadings comparing EFA and CFA. This is a result of not including of some items which loaded more than one factor (see Fig 4).

## General discussion

The presented results showed that the narrative technique of assessment of specific emotional scripts is reliable method in terms of inter-rater, test-rest reliability, and internal consistency. The presented findings of two studies allowed to identify the main components (grouped variables) of emotional scripts: Positivity (of actor in hate script), Negativity, Ambivalence, Rejection, and Indefinite picture. These components are varied in different emotions, however, they are relatively stable.

The results of two studies also documented the appropriate discriminant and prognostic validity of the technique. There have been showed differences in the components of emotional scripts between persons with Psychopathy and without this disorder. Furthermore, love, hate, and anxiety script components allow to predict psychopathic personality. In love scripts, prediction of Psychopathy scores is based on high Indefinite picture and Rejection, low in Ambivalence and Positivity. Positivity in love script includes (according to the exploratory factor analysis) positive assessment of the actor, actor’s positive emotions, and importance of love. Indefinite picture of love includes (basing on EFA) positive perceptions of a partner and unimportance of love. Then, low Ambivalence means that a person does not present at the same time positive and negative emotions/descriptions. Rejection of love includes actions against love and feeling/statements referred to unimportance of love. Thus, such characteristics/ components of love script in a psychopath inform us about their behavioural inconsistencies and incongruence of love. On one hand, a psychopath feels positively while assessing himself. When he focuses on himself, love seems to be positive and he feels positive emotions, and when he focuses on a relationship or a partner, he does not feel sure, his sense of security is low and he rejects love relationship. He expresses it by saying that love is unimportant. On another hand, when he focuses on himself, he highlights the significance of love. This complicated and incongruent picture of love can be revealed by the presented technique. The obtained data are consistent with findings in literature indicating that a description of love in persons with this disorder is focused on the narrator, his emotions, and his sense of security, while acknowledging the tremendous significance of the event [13, 14]. This is potentially associated with the excessive concentration on themselves and limited attention to others in persons with psychopathic personality [49, 50]. The data reported in the literature emphasized limited capacities for empathy and narcissistic tendencies in individuals with such disorders [14, 50]. The hate script in persons with Psychopathy encompasses significant characteristics such as high Ambivalence, high Indefinite picture of hate, high Positivity of actor, low Negativity, and low Rejection. These patterns of scripts explain notable percent of variance of the Psychopathy scores, i.e. 69%. This indicates that hate script’s patterns are particularly informative on emotional functioning of persons with psychopathic personality. They report in narratives to behave in a hate situation in an opposite way to typical, they seem to not perceive the negative valence of hate. This emotion is associated in psychopathic persons with contradictive tendencies, positive perception of self, and not full understanding of the hate situation. This is coherent with the opinion presented by Beck, Freeman and Davis that individuals with antisocial personality talk about themselves in a characteristic way, claiming they are kind, unique and amicable, even in negative emotional situation [13]. Contradicted by various real-life facts, the statement “I’m always easy to get along with” [activity directed at people, activity “towards”, see Fig 1] points to the speaker’s very strong convictions regarding their personal competences, and is associated, on the one hand, with positive self-esteem and on the other, with a lack of insightfulness in reflecting on their conduct [14, 50]. Affective deficits of this type are shown by the findings related to assessment of situations and their meanings; causes of incidents are described by individuals with psychopathy as insignificant, trivial or inconsequential [51]. This indicates a fixed tendency for formulating definitive statements (such as “all women. . .” or “men always. . .”) (see the examples of the narratives, section Procedure, Results in this paper). This feature seems to be associated with cognitive rigidity, with a propensity for inflexibility in perceiving the world [43, 49, 50]. While discussing the development of rigidity, it has been argued that aetiology of this feature is linked with a strong feeling of anxiety and a cognitive pattern which makes it impossible to search for more flexible solutions [49]. Researchers believe that development of the information processing system takes place in conditions associated with strong focus on a threatening situation and with a limited capacity to cope with it [52, 53]. The contents of hate scripts in individuals with psychopathic personality correspond to the information contained in the cognitive model of personality disorders proposed by Beck and associates [13]. The obtained findings show that it is typical for psychopathic personality to perceive the world in black-and-white categories, which is reflected in the belief “I am unique, kind, and always ready to agree”, and “the others are evil, mean and always. . .behave inappropriately”. Resulting from dysfunctional assumptions, key beliefs in a negative situation (i.e. involving hate) contribute to significant limitations in the capacities for drawing conclusions and for working out the conflict, because an individual with psychopathic personality does not see the problem [13, 43, 51, 54]. The contents of love and hate scripts are an illustration of numerous cognitive distortions and deficits in the emotional information processing in individuals with psychopathic personality [50, 51]. It is characteristic for them to inaccurately perceive situations faced by them; people with this disorder tend to exaggerate, minimise or generalise excessively. Furthermore, they filter out information in a specific way, as if to emphasize their competences and at the same time reject or ignore critical information [8, 9, 49]. Thus, the hate scripts have the attributes as Indefinite picture, Ambivalence, low Rejection, and Positivity of actor. The analysis presented here adds the insightful information of the content of emotional scripts. This allows to predict psychopathic behavior and dysfunctional emotionality. Thus, it lets to describe their emotional impaired functioning insightfully.

The patterns of anxiety script can also predict Psychopathy. The aspects as high Ambivalence, high Negativity, high Rejection, and low Indefinite picture of anxious situation are the most informative in terms of associations with psychopathic personality traits. These patterns are similar to the love and hate scripts in persons with high Psychopathy. It indicates that these persons are unable to understand the emotional situations, their valence and meaning, which is typical for the analysed disorder [8, 9,48–50].

The component of specific emotion scripts also allow to predict state and trait anxiety. The most informative on state anxiety is a component high rejection in love. Then, low rejection of hate, and high ambivalence and low positivity of anxiety, all of them predict anxiety as a trait. These findings are consistent with the general characteristics of anxious persons [33]. The components of anxiety script are the most predictive for the trait anxiety. The content of anxiety scripts reflects typical patterns of functioning of persons with trait anxiety [55]. The results clearly indicate that psychopaths are differentiated in terms of their experiencing anxiety. Among them there are individuals who experience anxiety in more intense way, other who experience at lowered level. In sum, they react in non-adequate way to stimuli and their processing of threat is impaired [56]. They possess different elements in the content of anxiety scripts.

### Advantages of the method of the emotional scripts assessment

The presented novel method provides useful information on emotional scripts beyond existing standard, traditional self-report measures. Persons with disorders are not aware of their dysfunctions, they have impaired insight in their emotions, and that is why while they complete traditional self-measures their results are distorted/false/or associated with artifacts. The presented technique allows avoiding this problem and it lets to describe their emotional impaired functioning as well as to predict dysfunctional emotional behavior. Two studies have been conducted with the use of the presented technique and it has been found that this method enables collection of informative data on romantic love, hate, and anxiety scripts in people with psychopathic personality and with trait anxiety. It has been highlighted that emotional narratives reflect the fundamental components of their affective scripts. The findings have provided insight into how these individuals may experience emotions and how they emotionally behave. The analysis adds the insights on the content of emotional scripts. This allows to predict psychopathic behaviors and dysfunctional emotionality. This comprehensive analysis shows the complex patterns of Psychopath emotionality, it is not focused on single characteristics as, for instance, self-measure techniques are. We were not intended to explain how single specific properties of scripts relate to Psychopathy. The purpose was to establish the configuration of markers/complex aspects of emotional scripts which let to describe and understand emotionality of persons with personality disorder.

## Conclusions

To recapitulate, the method comprises the following novelty elements: it introduces a technique for assessing scripts of specific emotions; the proposed technique is based on the narrative methodology (it is not a self-report technique); it allows to examine scripts related to three main emotions i.e. romantic love, hate, and anxiety, which are highly significant in psychopathology because people with disorders display specific emotion deficits; the technique does not refer to autobiographical memories; and finally the coding system was derived from a sample of adults representing general population. The system coding is not laborious. The reliability (test retest, inter-rater agreement), internal consistency, and validity (discriminant, predictive) have been established. In conclusion, the method presented here, designed for investigating scripts of specific emotions, is informative and brings a new perspective to clinical psychology and psychopathology.

### Limitations and future directions

Although the proposed technique is of value, it is not free of limitations. The main limitation is that it has been tested in a limited number of disorders, mostly in psychopathy. With regard to emotional problems it was tested only in relation to state/trait anxiety. The method has not been yet compared to other technique measured emotional scripts, however, it would be difficult because of lack of the identical techniques. Nevertheless, some aspects from other techniques can be compared to the presented here method. In sum, the construct and concurrent validities of the technique were not fully verified.

Future research should focus on a larger group of disorders, such as affective and others personality disorders, to better document discriminant validity. Possibly, it will require elaboration of new indicators as not each indicator included in a factor was confirmed in CFA. This is because some indicators loaded more than one factor. In order to achieve a goodness of fit parameters, some indicators have been removed from the factors. Possibly, another statistical analysis is needed, not CFA but rather k-cluster analysis to establish the potential configuration of factors. Then, the computational aspect, which would be important for diagnosticians, has not been yet expanded. There is also a need for improvement of a software to accelerate the coding process of qualitative data, data analysis, and allow for easier data replication. Scientists highlight that language is psychologically rich with information and they propose the measures allowing description of psychological characteristics [57].

## Supporting information

S1 Fig Scree plots EFA. (PDF)

S1 Table Inter-rater correlations (the intra-class correlations between three independent coders). (PDF)

S2 Table Intergroup comparisons between inmates with high Psychopathy (Psych), inmates with low psychopathy, and non-prisoners (n = 200). (PDF)

S3 Table Summary multiple regression analysis. (PDF)

S4 Table Test-retest Pearson’s correlations (between 1rst and 2nd measure, i.e. after four months). (PDF)
