# Supplementary material for: The novel narrative technique uncovers emotional scripts in individuals with psychopathy and high trait anxiety
Source: PLoS One. 2023 Mar 23;18(3):e0283391. doi: 10.1371/journal.pone.0283391 (PMC10045615; doi:10.1371/journal.pone.0283391)
Supplement: S1 Fig — (PDF) [file pone.0283391.s001.pdf]

S2 Figures. Scree plots EFA

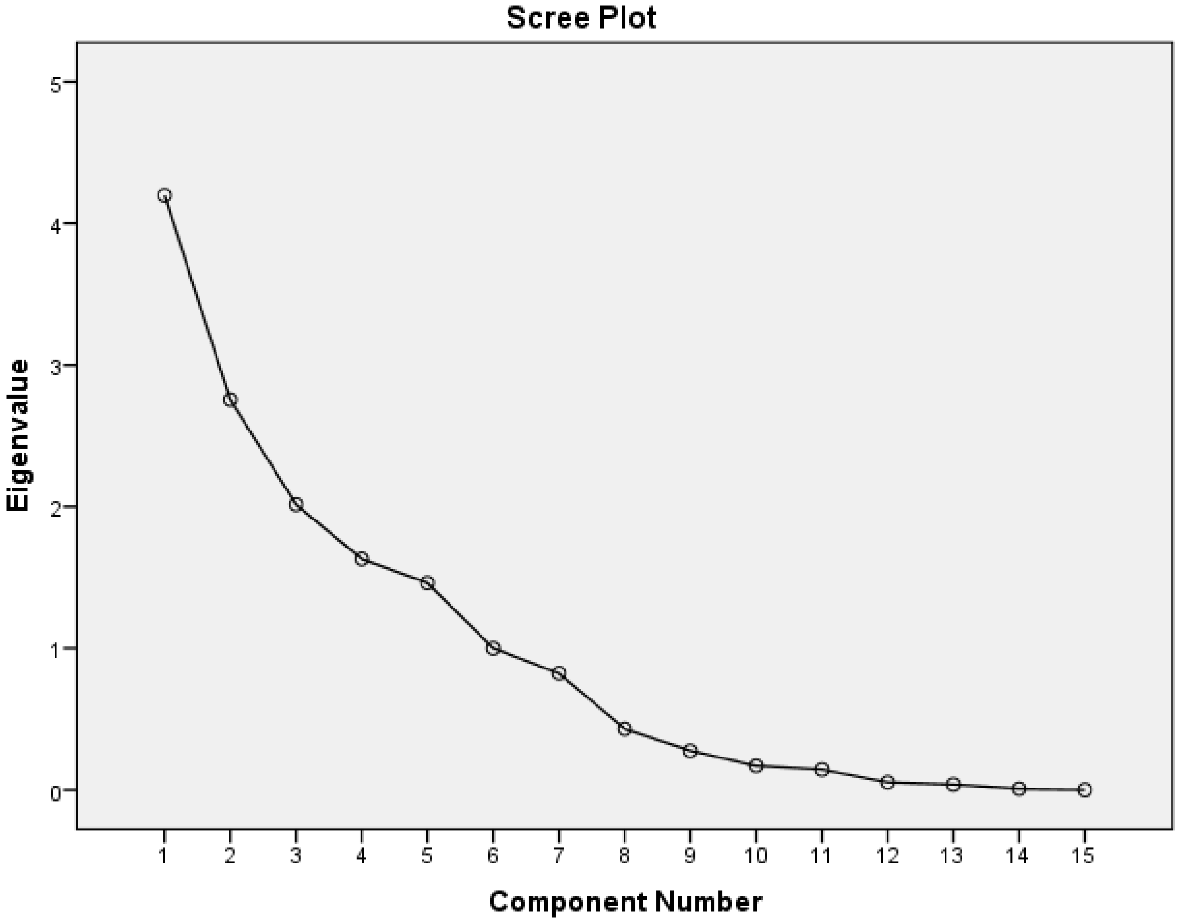

Fig. 1. Love: EFA – scree plot

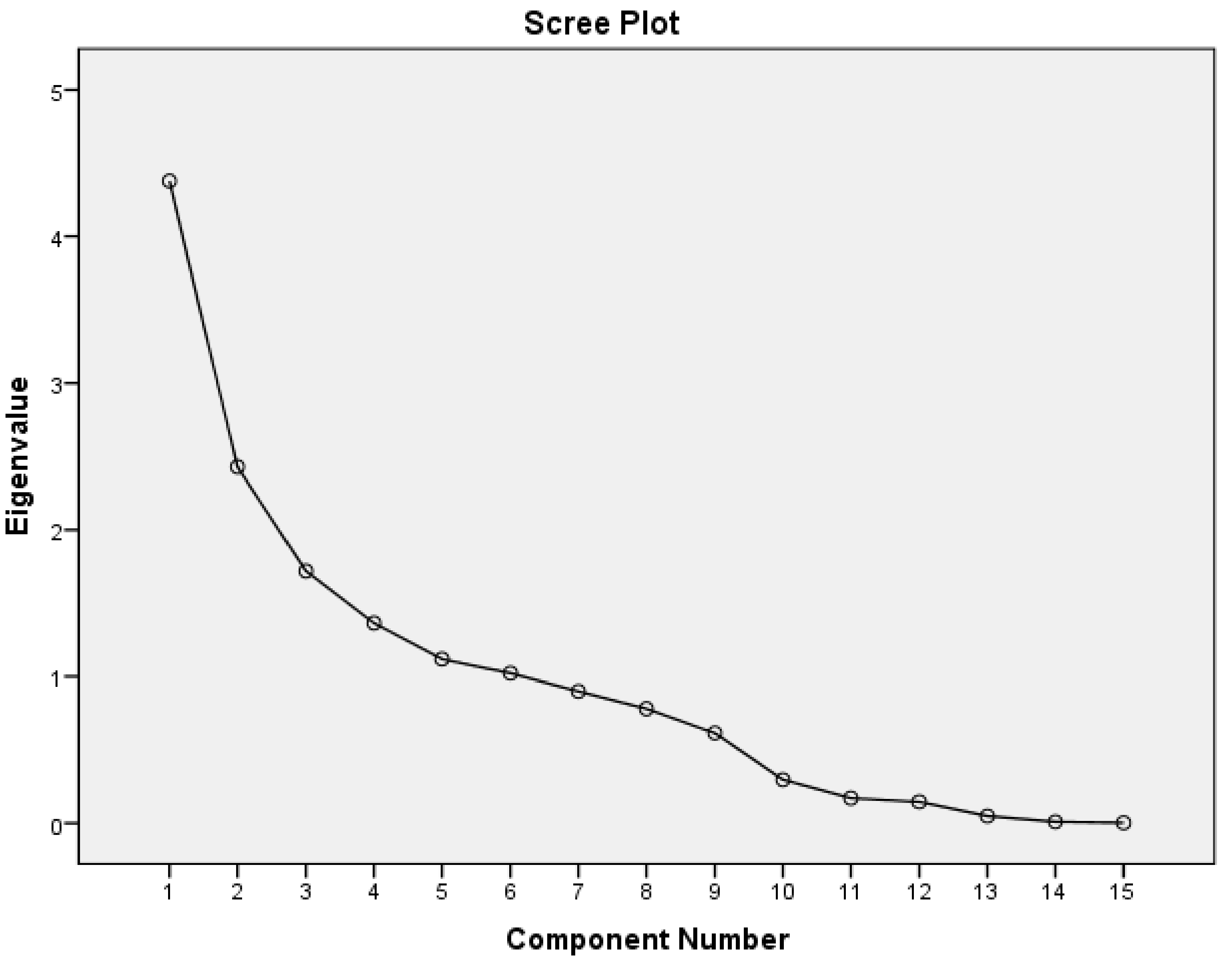

Fig 2. Hate: EFA – scree plot

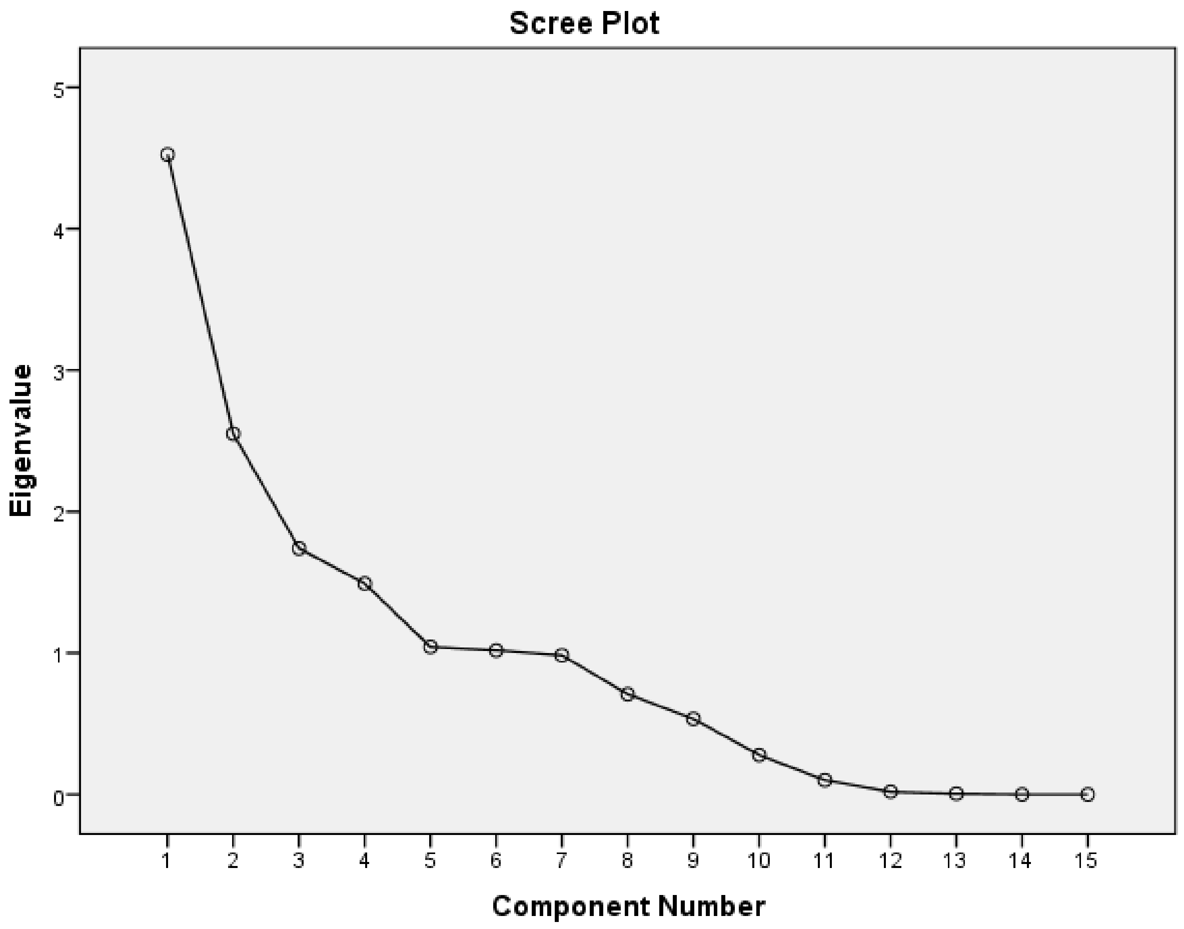

Fig. 3. Anxiety: EFA – scree plot
