# Supplementary material for: The novel narrative technique uncovers emotional scripts in individuals with psychopathy and high trait anxiety
Source: PLoS One. 2023 Mar 23;18(3):e0283391. doi: 10.1371/journal.pone.0283391 (PMC10045615; doi:10.1371/journal.pone.0283391)
Supplement: S1 Table — (PDF) [file pone.0283391.s002.pdf]

SUPPLEMENTARY MATERIALS

Table 1. The inter-rater correlations (the Intraclass Correlations between three independent coders)

**Variables**	**Love (*n*=200)**	**Hate (*n*=200)**	**Anxiety *n*=200)**
**Actor negative**	.90***	.93***	.97***
**Actor positive**	.91***	.93***	.95***
**Actor negative emotions**	.92***	.93***	.95***
**Actor positive emotions**	.96***	.94***	.95***
**Partner negative**	.95***	.93***	.94***
**Partner positive**	.93***	.92***	.92***
**Partner emotions negative**	.96***	.94***	.94***
**Partner emotions positive**	.94***	.94***	.95***
**Actions towards**	.90***	.90***	.91***
**Actions from away**	.91***	.84***	.83***
**Actions against**	.84***	.84***	.83***
**Important**	.85***	.85***	.85***
**Unimportant**	.84***	.84***	.83***
**Positive ending**	.83***	.84***	.84***
**Negative ending**	.84***	.83***	.84***

***p<.001
