# Supplementary material for: The novel narrative technique uncovers emotional scripts in individuals with psychopathy and high trait anxiety
Source: PLoS One. 2023 Mar 23;18(3):e0283391. doi: 10.1371/journal.pone.0283391 (PMC10045615; doi:10.1371/journal.pone.0283391)
Supplement: S2 Table — (PDF) [file pone.0283391.s003.pdf]

## SUPPLEMENTARY MATERIALS

**Table 5. Intergroup comparisons between inmates with high Psychopathy (Psych), inmates with low Psychopathy, and non-prisoners (*n* = 200)**

| | **Groups** | ***M*** | ***SD*** | ***F*** | ***p*** |
|---|---|---|---|---|---|
| **Love Indefinite** | Inmates high Psych | 19.77 | 2.83 | 7.70 | .001*** |
| | Inmates low Psych | 16.56 | 1.06 | | |
| | Non-prisoners | 13.10 | 2.03 | | |
| **Love Positivity** | Inmates high Psych | 15.46 | 4.15 | 12.02 | .000*** |
| | Inmates low Psych | 15.60 | 2.75 | | |
| | Non-prisoners | 19.13 | 3.49 | | |
| **Love Negativity** | Inmates high Psych | 8.13 | 1.48 | 5.98 | .003** |
| | Inmates low Psych | 7.20 | .40 | | |
| | Non-prisoners | 7.41 | .81 | | |
| **Hate Ambivalence** | Inmates high Psych | 8.85 | 1.40 | 66.71 | .000*** |
| | Inmates low Psych | 5.03 | .64 | | |
| | Non-prisoners | 5.33 | 1.15 | | |
| **Hate Negativity** | Inmates high Psych | .64 | 1.71 | 64.20 | .000*** |
| | Inmates lowPsych | .95 | .86 | | |
| | Non-prisoners | 2.37 | .69 | | |
| **Hate Rejection** | Inmates high Psych | 2.00 | .00 | 24.04 | .000*** |
| | Inmates low Psych | 2.38 | .75 | | |
| | Non-prisoners | 3.20 | .49 | | |
| **Anxiety Ambivalence** | Inmates high Psych | 7.31 | 2.17 | 9.86 | .000*** |
| | Inmates low Psych | 6.18 | .75 | | |
| | Non-prisoners | 5.80 | 1.17 | | |
| **Anxiety Negativity** | Inmates highPsych | 3.11 | 1.21 | 44.483 | .000*** |
| | Inmates low Psych | 7.20 | 2.42 | | |
| | Non-prisoners | 8.41 | 4.79 | | |

**$p$<.01, ***$p$<.001
