# Supplementary material for: The novel narrative technique uncovers emotional scripts in individuals with psychopathy and high trait anxiety
Source: PLoS One. 2023 Mar 23;18(3):e0283391. doi: 10.1371/journal.pone.0283391 (PMC10045615; doi:10.1371/journal.pone.0283391)
Supplement: S3 Table — (PDF) [file pone.0283391.s004.pdf]

SUPPLEMENTARY MATERIALS

Table 6. Summary of multiple regression analysis

Predictors	B	SE	Beta	*t*	*R*	R^2	$F_{(15, 183)}$
**Love Ambivalence**	-.995	.127	-.472	-7.832***	.92	.85	53.46***
**Love Indefinite**	1.846	.238	.876	7.745***			
**Love Positivity**	.122	.128	.058	.950 ns			
**Love Rejection**	.244	.073	.116	3.359***			
**Love Negativity**	.245	.130	.116	1.885 ns			
**Hate Ambivalence**	-1.166	.266	-.553	-4.385***			
**Hate Negativity**	-.571	.174	-.271	-3.289***			
**Hate Indefinite**	-.115	.203	-.055	-.568 ns			
**Hate Positivity**	1.139	.126	.540	9.061***			
**Hate Rejection**	-.123	.112	-.058	-1.103 ns			
**Anxiety Ambivalence**	.790	.179	.375	4.415***			
**Anxiety Negativity**	-1.230	.197	-.583	-6.242***			
**Anxiety Indefinite**	-.917	.198	-.435	-4.643***			
**Anxiety Rejection**	1.187	.143	.563	8.297***			
**Anxiety Positivity**	-.049	.079	-.023	-.627 ns			

***p<.001, ns – non-significant
