# Supplementary material for: The novel narrative technique uncovers emotional scripts in individuals with psychopathy and high trait anxiety
Source: PLoS One. 2023 Mar 23;18(3):e0283391. doi: 10.1371/journal.pone.0283391 (PMC10045615; doi:10.1371/journal.pone.0283391)
Supplement: S4 Table — (PDF) [file pone.0283391.s005.pdf]

SUPPLEMENTARY MATERIALS

Table 7. Test-retest Pearson's correlations (between 1rst and 2nd measure, i.e. after four months)

**Variables**	**Love (*n*=280)**	**Hate (*n*=280)**	**Anxiety *n*=280)**
**Actor negative**	.82***	.82***	.81***
**Actor positive**	.78***	.82***	.82***
**Actor negative emotions**	.78***	.83***	.84***
**Actor positive emotions**	.78***	.76***	.76***
**Partner negative**	.76***	.77***	.76***
**Partner positive**	.84***	.84***	.84***
**Partner emotions negative**	.84***	.84***	.83***
**Partner emotions positive**	.85***	.95***	.95***
**Actions towards**	.80***	.80***	.81***
**Actions from away**	.81***	.85***	.85***
**Actions against**	.83***	.83***	.83***
**Important**	.82***	.82***	.82***
**Unimportant**	.80***	.80***	.80***
**Positive ending**	.77***	.76***	.76***
**Negative ending**	.76***	.76***	.76***

***p<.001
